# Sculpting the good surgeon or excising the bad one: How clinical teachers could perpetuate attrition in surgical residency programmes

**DOI:** 10.1111/medu.15557

**Published:** 2024-10-20

**Authors:** Arlen Astrid Rada‐Estarita, María Camila Rincón‐Ortiz, Oscar Geovanny Hernández‐Rodríguez, Francisco Manuel Olmos‐Vega

**Affiliations:** ^1^ Clínica Iberoamericana and Clínica Portoazul Barranquilla Colombia; ^2^ CAFAM Bogotá Colombia; ^3^ Hospital Universitario San Ignacio Bogotá Colombia; ^4^ Pontificia Universidad Javeriana Bogotá Colombia

## Abstract

**Introduction:**

Attrition in surgical residencies remains a significant issue, with traditional research focusing mainly on individual and programme factors. This study explores the role of clinical teachers (CTs) in influencing attrition rates. CTs are essential in moulding residents' training, serving both as enablers of workplace learning and guardians of their medical fields.

**Methods:**

We employed a hermeneutic phenomenology framework to understand the sociocultural impacts on attrition. Data were collected through semi‐structured interviews involving 19 CTs, 3 residents who left the programme and 2 who underwent remediation, following a six‐step hermeneutic phenomenological analysis process.

**Results:**

The entrenched ‘good surgeon’ narrative within the department demanded selflessness and total dedication, which CTs reinforced, thereby normalising a rigorous and challenging environment. This has led to attrition when residents fail to meet these challenges or choose to disengage from the system. We illustrated that CTs were pivotal in perpetuating these expectations, contributing significantly to resident attrition.

**Conclusions:**

CTs played a crucial role in resident attrition by enforcing a stringent cultural norm within surgical training programmes. Addressing this issue requires a visible change in CTs' role to foster a more supportive educational environment. Emphasising the beneficial aspects of the ‘good surgeon’ narrative and mitigating its adverse impacts is essential for reducing attrition rates and assisting all residents, including those facing challenges, in successfully completing their training.

## Abstract 2

New research uncovers how clinical teachers' "good surgeon" narratives contribute to high attrition rates in surgical residencies. #MedEd #SurgicalResidency #Attrition

## 1 INTRODUCTION

The prevalence of attrition in surgical residencies continues to be a significant concern, characterised by its persistent high rates.^1^, ^2^ Historically, research investigating this issue has primarily centred on individual and programmatic factors, exploring the characteristics and circumstances of the residents themselves,^3^, ^4^ as well as the structural elements of the residency programmes contributing to the problem.^5^, ^6^ However, there is a notable gap in the literature regarding the influence clinical teachers (CTs) might have on this issue. CTs are pivotal in shaping residents' training experiences, acting both as facilitators of engagement with workplace learning opportunities and as custodians of their medical specialties.^7^, ^8^ Thus, comprehensively understanding their influence on attrition is vital, offering potential strategies to reduce its prevalence effectively.

Attrition among general surgery postgraduates represents a pervasive issue with far‐reaching implications. The phenomenon exhibits regional variability, with particularly higher rates in Pakistan, the United States, Canada and the United Kingdom, where attrition rates have soared to as high as 38.6%.^9^, ^10^ In contrast, the Netherlands and West China report comparatively lower rates.^11^, ^12^ The repercussions of attrition are multifaceted: residents who depart from surgical programmes frequently experience demoralisation, haunted by the spectre of squandered time and resources.^13^, ^14^ Concurrently, the residency programmes grapple with amplified costs related to selection and training, intensified by the challenge of filling vacancies left by departing residents.^15^, ^16^ This scenario underscores the urgency for a comprehensive understanding of attrition dynamics and the formulation of strategic interventions tailored to mitigate these losses within the global context of surgical education.

Research on attrition has centred on the individual and programmatic perspectives of the problem, often with contradictory findings. On an individual level, demographic variables such as sex (2), age (17), ethnicity (3), marital status and pregnancy have been explored,^17^ but no definitive conclusions have been reached due to varying results. Similarly, resident traits such as behavioural styles, psychological profiles and grit have been considered.^13^, ^18^, ^19^ Academic performance,^20^ personal interests^21^ and financial concerns^22^ during training have also been examined without reaching a consensus on their impact on attrition. On the programmatic side, early years of training—particularly in the first and second years—have been identified as critical for attrition,^1^ which appears to be higher in military programmes, prestigious institutions and programmes with a long‐standing tradition of attrition.^3^, ^5^ The size of the residency programme and the introduction of working hour regulations have been discussed, with mixed conclusions regarding their influence on attrition rates.^23^ In summary, despite extensive research, a clear understanding of the drivers behind attrition remains elusive, signalling that the answer does not solely rest within the individual or programmatic perspective.

An in‐depth sociocultural exploration holds promise for enhancing our comprehension of the multifaceted nature of attrition in surgical residencies, potentially unveiling the deeper, more complex dynamics at play. Sociocultural research conducted on surgical programmes found that the surgical training environment is marked by its rigorous nature, hierarchical structure and the competitive, high‐demand culture that prevails in Western education systems and those modelled after them.^24^, ^25^, ^26^ While these elements are often considered essential for preparing residents for the demanding nature of the surgical profession by fostering resilience and a strong awareness of the consequences of errors, they also create an environment conducive to bullying, discrimination and harassment.^27^, ^28^ Furthermore, the intense demands of training, prioritising workload and efficiency, often at the expense of educational quality, could contribute significantly to attrition rates.^14^ Underrepresented minority and female surgical residents, in particular, face distinct and more challenging training experiences, often burdened by limited leave options, gender‐based discrimination and a lack of role models and support.^29^, ^30^ They are frequently required to demonstrate resilience in a male‐dominated environment, often enduring bullying, harassment and exclusion.^31^, ^32^ This toxic educational culture and the resultant resident burnout^33^—a potential antecedent of attrition—highlight the urgency of addressing these sociocultural factors. However, current research has yet to thoroughly explore the influence of training culture on attrition, particularly the role of CTs within this context—a gap that, if addressed, could reveal pivotal insights into attrition and inform the development of more supportive educational environments. Therefore, our research question is as follows: how do CTs influence the attrition phenomenon in surgical residency programmes?

## 2 MATERIAL AND METHODS

### 2.1 Research design

To explore the role of CTs in residents' experiences of attrition, we employed a hermeneutic phenomenology approach, which is designed to deeply engage with the lived experiences of participants and the contextual factors that shape those experiences.^34^ This methodology enabled us to focus on how leavers and CT experience the attrition phenomenon, allowing us to uncover the complex, often hidden dynamics that contribute to the decision to leave a residency programme. Central to this approach is the emphasis on participants' narratives, which serve as a primary data source to illuminate the personal and shared meanings they attribute to their experiences.^35^ These narratives are crucial in capturing the richness and diversity of the residents' experiences, providing insight into how individuals perceive, interpret and respond to the cultural norms and expectations of their training environment.^36^

Hermeneutic phenomenology does not attempt to distance the researcher from the subject of study; instead, it embraces the notion that researchers' personal experiences and backgrounds are intertwined with the interpretive process.^35^ This perspective allows for a deeper exploration of the more nuanced aspects of the participants' stories, recognising that the researchers' insights and reflections contribute to a more comprehensive understanding of the studied phenomena.^37^ During the interviews, we asked participants to share detailed accounts of their experiences with attrition, focusing on the contextual factors that shaped these experiences. By doing so, we aimed to elicit rich, descriptive narratives that could reveal the interplay between individual experiences and the broader cultural environment of surgical training programmes.

Our reflective engagement with these narratives and our own experiences with attrition allowed us to connect more deeply with the participants' stories. This process informed our interpretations and enhanced our empathy and understanding, fostering a richer, more layered analysis of how CTs and the sociocultural context of surgical training influence resident attrition. By foregrounding the participants' narratives, we were able to illuminate the complex dynamics at play, providing valuable insights into the cultural factors that contribute to attrition in surgical residency programmes.

### 2.2 Context

We conducted this study at one general surgery programme in Colombia, an accredited residency programme that admits six residents per year. All surgeons affiliated with this department also serve as CTs in the residency programme, supervising not only surgery residents but other discipline residents and undergraduate students. Of note, 90% of the surgeons in this department were trained in the same programme. This programme has an attrition rate of 25%. In Colombia, residency programmes last from 3 to 5 years after an initial 6‐year undergraduate education that includes a 1‐year internship. To apply to a residency programme, newly graduates must make a social service period, often in rural areas, where they practise as general physicians with government salaries. Residency training features 2‐ to 3‐month rotation blocks across various specialties. Each programme centres around a principal learning institution but also integrates external rotations for specialised knowledge. This specific programme has rotations in major hospitals in Bogotá and Medellin. Residents undertake a broad range of clinical duties under supervision, advancing through a model of progressive entrustment. Their workplace learning is enhanced by activities such as journal clubs, morbidity and mortality rounds, interdisciplinary discussions and small‐group sessions.

### 2.3 Data collection and analysis

Adopting a purposeful sampling strategy, we selected CTs from the surgery department with various years of experience (both as CTs and surgeons), various levels of training and gender. Most of the CTs who agreed to participate were also residents in the same department, so we also had access to their experiences as trainees (see Table 1). Additionally, we invited surgeons who had difficulties finishing the programme but managed to graduate and all the leavers from the last 5 years. We made a strategic decision to interview only CTs and residents who had left the programme for several reasons. First, the majority of the current CTs (90%) had themselves been residents within the same programme. This dual perspective allowed us to explore their experiences both as former residents and as current faculty members who shape the training environment. Their reflections on their past experiences as residents, combined with their insights as educators, provided a unique longitudinal view of how the culture of the programme has been perpetuated or evolved over time. Additionally, interviewing residents who had left the programme gave us direct insight into the factors contributing to attrition. These individuals were able to speak openly about their reasons for leaving without the potential pressures or constraints that current residents might feel when discussing sensitive topics like dissatisfaction or challenges within the programme. Leavers provided candid perspectives that could have been difficult to obtain from those still in the program, who might hesitate to share their concerns for fear of repercussion or jeopardising their standing in the residency.

**TABLE 1:** Clinical Teachers' characteristics

Characteristics	Participants
Age	
25–35	4
36–45	8
46–55	3
>55	4
Gender	
Female	4
Male	15
Specialty	
General surgeon	9
Fellowship	10
Year of practice	
0–5	6
6–10	4
10–15	2
16–20	2
>20	5
Formal training in education	
Yes	2
No	16
In Progress	1
Year of clinical teaching experience	
0–5	6
6–10	4
10–15	2
16–20	5
>20	5

We conducted 24 semi‐structured interviews of approximately 90 min, comprising 19 CTs, 3 residents who left the programme in the past 5 years and 2 residents who completed the training despite undergoing a remediation process. Specifically, the leavers choose to leave the programme. We chose individual interviews to facilitate open discussions on attrition, recognising its sensitivity; this approach allowed participants to freely express their thoughts and feelings about the phenomenon.^38^ Interviews were conducted by MRO, ARE and OHR. MRO and ARE conducted interviews with all leavers, residents who struggled to complete the programme and junior CTs as they did not have any power relationship with them. OHR conducted interviews with intermediate and senior CTs. The interview guides were constructed to explore participants' lived experiences while underpinning the narratives used to interpret the attrition phenomenon, as detailed in Appendices S1 and S2.^37^ Each interview was recorded and transcribed for analysis. The analysis employed a hermeneutic process, enhancing our interpretation of the phenomenon under study, as outlined by Ajjawi and Higgs.^38^

This data analysis process encompasses a series of interconnected stages to distil and interpret qualitative data (See Table 2). This interpretive analysis was supported by ATLAS.ti (atlasti.com, ©2023 ATLAS.ti Scientific Software Development GmbH). Data collection and analysis continued iteratively until we achieved theoretical sufficiency.^39^

**TABLE 2:** Stages of the hermeneutical analysis

Analysis stage	Activities performed
Immersion	ARE, MRO and OHR read all the interviews, engaging deeply with the data to form a preliminary understanding or ‘sense’ of the information gathered. FOV read only a portion of the interviews
Understanding	ARE, MRO and OHR engaged in a first round of coding to create first‐order constructs. FOV reviewed the process and give suggestions to improve them. These constructs were grounded in participants own language and perspectives
Abstraction	All researchers engage in a second level coding stage to enrich the initial interpretations that entailed grouping the most salient first‐order constructs in second‐order constructs
Synthesis	All researchers oscillate between the literature, the data and previous analyses to construct themes and unearth meanings that participants may not have explicitly expressed, constant comparing the constructed themes with new and already collected information
Illumination	In this stage, we reconstructed and reconciled participants' narratives with the researchers' interpretations, shedding light on the data's journey and surfacing key discoveries, attending to personal and interpersonal reflexivity
Integration	We tested and refined the final themes to craft a cohesive final analysis, ensuring the findings were robust and representative of the studied phenomena. This included comparing the final themes across all the interviews and asking participants to give us feedback on the constructed results

### 2.4 Reflexivity

Reflexivity, a key concept in qualitative research, refers to the ongoing process of critically examining how researchers' backgrounds, subjectivity and positionalities shape the research process and the constructed results.^40^ In line with the hermeneutic phenomenology tenets, we recognised that the subjective perspectives of team members, particularly those with direct or related experiences within surgical departments, would inevitably influence our interpretations of the data. The research team consisted of a chief nurse (MRO), one general surgeon (ARE), one vascular surgeon (OHR) and an anaesthetist (FOV). OHR was the only team member who was part of the general surgery department where we conducted the study. We addressed his personal reflexivity through a self‐interview using the questioning guide we developed for CTs.^40^ The interview was recorded and transcribed to be actively compared with those of the participants. Additionally, we continually discuss his privileged position within the department we were exploring, addressing the effect this might have had on his interpretation of the data. ARE—the other general surgeon of the research team—completed an autobiographical narrative focusing on the attrition experiences they lived with fellow residents or as part of their CT life.^41^ We held regular meetings to discuss the constructing findings while attending to our team's interpersonal reflexivity.^42^ By addressing reflexivity at multiple levels, we were able to enhance the confirmability and depth of our analysis, ensuring that our subjectivities contributed meaningfully to a richer, more nuanced understanding of how CTs influence attrition.

### 2.5 Ethical considerations

We received ethical approval from the institutional review board of the institution where the surgical department was part. Informed consent was requested by two of the researchers, who were free of any real or potential conflict of interest with participants, as they were not part of the department where the study took place. Likewise, these two researchers conducted interviews with the residents who dropped out of the programme to ensure their anonymity. Data were deidentified, and, given the topic's sensitive nature, we redact or decouple data that could link participants to their data, including personal details, time and chronology details and relationships within the events recounted during the interviews. These anonymised transcripts were stored in a OneDrive folder shared by the researchers and accessed through a password.

## 3 RESULTS

Within the general surgery residency programme, a dominant narrative delineated the attributes of an ideal surgeon, emphasising selflessness and an unwavering commitment to patient care. These attributes were not only celebrated but also expected in physicians aspiring to become surgeons. CTs used their agency to actively reinforce this narrative, affirming residents who embody these ideals or marginalising those perceived as lacking the requisite qualities, effectively pushing them out of the programme. Daily practices, steeped in relentless pressure, serve as a crucible to determine residents' suitability for the surgical profession. Those who meet these rigorous standards gain the authority to exclude peers who fall short, invoking the narrative of the surgeon's social responsibility to the community. We also found a tension among the data, as leavers and struggling residents pose significant weight on CT actions on the phenomenon of attrition, while CT seemed to be unaware of this inference. In the following themes, we elaborate on these findings. The code following each quote distinguishes its source, with ‘F’ denoting faculty and ‘R’ signifying interviews with residents facing challenges or those who have left the programme.

### 3.1 The good surgeon narrative

The ‘good surgeon’ narrative, deeply embedded in the surgical department, prescribed a set of expectations for ideal surgeon traits. It valourised self‐sacrifice and unwavering patient dedication as hallmarks of excellence. Moreover, the ideal surgeon was portrayed as possessing a high tolerance for frustration, performing optimally under the high‐pressure demands typical of the surgical environment. Expected to embody stoicism, the surgeon should navigate through hardships and setbacks uncomplainingly, maintaining a facade of composure and enduring frustrations privately, without burdening others. The narrative emphasises the social responsibility of teachers to ensure that the surgeons they train are competent and do not pose a risk to patient safety. This narrative is captured in the following illustrative quote:

I feel that many times, one of the things that inspired us was that it was a difficult discipline [general surgery], […], it was a discipline where high demands had to be met and implied a great challenge of self‐improvement […], then the programs have lost the ability to […] tell the students that […] this is a difficult specialty where sacrifices have to be made in medical practice, […] I think that surgery has the stamp of being a demanding profession, to the point of sacrifice, and all this in order to serve the patients, […] it is not something innocuous, […] it's about serving the community, it's about people who could make an effort beyond themselves. (Participant F34)

### 3.2 The cycle of pressure and testing

Drawing on the ‘good surgeon’ narrative, CTs wielded their power to assess residents' alignment with these expectations. They employed their authority to apply continuous pressure through challenges designed to test the residents. When residents fail to meet the expectations of the testing and challenge system, they become stigmatised. Subsequently, residents felt they were subjected to even more demanding challenges and responsibilities without receiving sufficient support or resources to surmount their obstacles. CTs viewed a resident's mistake as a chance to teach through embarrassment, employing punitive and public displays to prevent similar mistakes in the future. The subsequent quotes provide insights into how this pressure has been applied:

So I think they [the residents] are less willing to sacrifice many of the things that we sacrificed in another era because they probably see that it's not their lifestyle. […] a lot of the faculty, even the younger ones […], give up all the other things in life in favor of surgery, and that if you're not in there 100% of the time, you don't like surgery, and if you want to rest or sleep it's not acceptable. (Participant F17)

Mortality rounds are a time of, I would call it, learning to be humble. […] Because you're picked out of all the residents for having made a mistake to expose that mistake, and the ways in which the mistake can be remedied, that I think is the academic end. However, the endgame is how they [the CTs] make him feel so bad and how everyone is going to feel so bad about that mistake that no one is going to do it again. (Participant F26)

Succeeding in these challenges leads to the adoption of the prevailing narrative, which in turn sustains the existing system. In this system, residents then apply the same pressures to their juniors, a cycle reinforced by the residents' increasing high positionality and power as they progress, potentially up to becoming CTs. The following quote clearly illustrates how senior residents continue the cycle of pressure and mistreatment upon junior ones:

Well, let's say passing a daily round, […] sometimes teachers asking things that were not relevant for a round. We had senior residents at that time that were worst, the tyranny and mistreatment of the senior R to the little R was tolerated, then it was something very difficult to manage, they would ask the resident for a blood result upon entry from a patient that has been hospitalized for a month, why would they ask me this? (Participant F70)

The pressure and challenge system is also rooted in the persistent belief that residents should be regarded as surgeons, not mere students. For instance, there is frequent mention of ‘surgical insight’, a nebulous skill that even CTs find challenging to articulate yet is considered intrinsic and unteachable. This belief serves to limit CTs own perception of their role in the attrition process, attributing residents' struggles to a lack of ‘surgical insight’—a quality they believe cannot be imparted—as described in the following quote:

Even though the man [the resident] tries hard, studies, and is disciplined, he is not able to realize that he has failed to figure out that reasoning, that expertise. Let's say that insight, you be suspicious and say, hey, this man [the patient] has something that I would like to solve, something serious. Yes, and that's very difficult to convey. And there are people that I say have it, and there are people that don't have it. That is something that I have not been able to explain to myself, why there are people who have that intuition, let's say, to achieve, to connect, and that intelligence. To be able to connect the information and achieve an appropriate clinical reasoning and those who do not, how to make them realize that they do not have it, without them feeling that it is something that is the system against them. It's just something that they lack. (Participant F35)

This notion further supports the view that attrition issues stem from flawed selection processes. CTs contend that residents who leave the programme either fail to properly evaluate the rigours they will face or lack the essential resolve required for the challenges of surgical training, as described in the following quote:

First, they are not completely convinced or informed about what the program is about and what they are going to do as surgeons, about the lifestyle imposed by the academic activity as a resident, and it is serious because they are not ready to endure it. […] and then one does not know at what point in the selection that flaw occurred, but I think that more than anything is the interview, […] it seems to me that in the interview there is much failure, it seems to me that many things are not explored about their personal life, their professional life, even about their geographical origin and then it is when they arrive and face a drastic life change that it hits them, and they are not prepared. (Participant F23)

### 3.3 Attrition as a consequence of failing the ‘good surgeon’ ideal

Within this department, attrition is seen as a resident's issue. Residents leave because they either subscribe to the ‘good surgeon’ narrative but fail to meet the CTs' demands, or they choose not to engage with this narrative, refusing to subject themselves to the rigorous testing regime. Those who aspire to the ‘good surgeon’ ideal but fall short in the pressure cooker of tests and challenges face confusion and do not understand where they falter, which leads to feelings of powerlessness and insecurity, as described in the following quote:

There was a central venous catheter programmed (…). So, I remember that I had read the technique very well and reviewed it because a few days before, I had not managed to pass a central venous catheter. So I reviewed the technique again, and in fact, my intention on entering the OR was a bit like redeeming myself (…). I went in, and as soon as I got the patient settled in, [the TC] started to disapprove of absolutely everything I did (…). And all of this in a tone, well, in a very high pitched tone. I was obviously uncomfortable. Basically, [the TC] didn't teach me anything or guide me in any way, the only thing he did was question and reproach absolutely every one of my movements (…) When I left the procedure, I said to the teacher: I need you to tell me what I was doing wrong (…), and the teacher gave me a very terse answer that he couldn't teach me how to feel when passing the catheter. That day, I decided to leave. (Participant R64)

They made me doubt about absolutely every aspect of me; they made me doubt my intellectual capabilities; about my personality and about whether my traits that I, I mean I, I had many, I have many personality traits that I consider valuable and I feel that in the department they were not only doubted but put to the test, I feel that they did a destruction of my self‐esteem. (Participant R64)

On some occasions, difficulties in passing the tests were related to economic difficulties and family problems that prevented residents from adapting to the demanding environment of the specialty. While these problems were inherent to the resident, unfortunately, in many cases, any help was hindered because some CTs were unaware of the resident's at‐risk situation. What is worst, even those who sought help were socially punished, as the following described in the following two quotes:

For example, my colleague who left after 3 days [in residency], his personal situation was that his mother had recently committed suicide, I never saw any situation of empathy [..] one of the most important people in his life just died, in a supremely violent condition, let's not make him start on the surgical ward, which is the heaviest rotation, let's make him start with nutritional support [which might be easier], or OK, if he must start on the surgical ward, what can we do to help him, how can we make a contribution? What I heard from him when he came back was that they made him start in the surgical ward. ‘Take it or leave it’, they said to him. (Participant R55).

But it's not frowned upon either, because if you seek help or if you go, if you know you're feeling bad or whatever, then it's also frowned upon, so it's not just that there is no time and that, but also that it was something socially punished. (Participant R16)

As previously mentioned, some residents chose not to conform to the department's expectations, unwilling to subject themselves to the tests and pressures, or upon reflection, recognised that a career in surgery was not their true calling. This self‐awareness led them to the decision to leave the programme, a choice they made independently as portray in the following quotes:

I realize that it wasn't even the path that was going to make me genuinely happy, because, as I told you, I'm interested in attending children, they are supremely different, that is, everything is completely different. (Participant R55)

So I feel that the one who enters and leaves is because he didn't like it. Because one of them studied radiology, the other one internal medicine, the other one finished something that has nothing to do with medicine. The other one is still a general practitioner, so it is very difficult, very difficult, let's say. Yes, to leave one of those positions. But I feel that it opens their eyes to say that this is not my thing and that is why they leave. (Participant F66)

## 4 DISCUSSION

In the general surgery residency programme, there is a prevalent narrative defining what makes a good surgeon—self‐sacrifice and complete devotion to caring for patients. These qualities are not merely applauded; they are requisites for those on the path to surgery. CTs exercise their influence to support this narrative, validating residents who reflect these values while sidelining those who do not measure up, thus excluding them from the professional community. The everyday environment, rife with intense pressure, acts as a proving ground for assessing residents' fit for the demanding surgical field. Successfully navigating these stringent expectations empowers residents to decide who among their peers does not belong, reinforcing the narrative that being a surgeon is not just a job but a societal duty.

In this study, we disentangled sociocultural factors impacting the attrition phenomenon in surgical programmes. The influence of culture could explain the contradictory findings in studies that focused on residents' individual characteristics or on programmatic factors.^1^, ^2^, ^3^, ^5^, ^17^, ^21^ First, attending to the sociocultural context allowed us to understand the role that social interactions among the main actors of this programme might have in creating and perpetuating a culture that facilitates resident attrition. By focusing on residents' individual characteristics and programmatic elements, we missed the big picture, failing to provide a clear answer about what perpetuates the issue. Second, because much of the literature sees attrition as a result of flaws in individual residents, this view may foster the perception that the role of CTs is limited. However, our study suggests that they have a pivotal role in perpetuating a culture that places its residents on the edge with relatively little opportunity for remediation or help. The combination of a narrative that assumes residents as surgeons, a system of constant pressure and challenges, as well as the perception that the responsibility for attrition falls on the student are sociocultural factors that propitiate such phenomenon.

These elements could create a toxic culture that privileges humiliation, shame and mistreatment in medical training, factors that have significant detrimental effects on both residents' performance and patient outcomes.^43^ Studies reveal that shame and feelings of inadequacy can erode a resident's resilience and increase the likelihood of burnout and mental distress.^44^ Such emotional states are linked to poorer clinical performance, reduced empathy and compromised patient safety.^32^, ^43^ Furthermore, a toxic environment where residents feel undervalued or humiliated could stifle open communication and lead to unreported errors, compromising patients' safety​. To enhance performance and patient safety, surgical education must shift from a punitive culture to one emphasising constructive feedback, emotional support and non‐technical skills, such as teamwork and communication, as core competencies.

Our study sheds light on the relationship between the surgical teaching culture and the phenomenon of attrition, a missing link in previous literature.^14^ Research on the social and cultural aspects of surgical training has revealed that such programmes are typically characterised by their strictness, hierarchical organisation and intensely competitive nature.^24^, ^25^, ^26^ These factors are considered crucial for preparing residents for the rigours of the surgical field, as they promote resilience and a critical understanding of the importance of avoiding mistakes.^24^ However, this same environment can also foster negative behaviours like bullying, discrimination and harassment.^27^, ^28^ We link this previous literature with the phenomenon of attrition by describing how CTs create an environment of intense pressure by leveraging narratives of concern about the well‐being of their patients, a culture that is perpetuated by others in the environment. By arguing that they are putting patients first—a worldwide priority in health care—CTs justify a sociocultural milieu that puts exaggerated demands on residents' performance without providing them with the necessary tools to navigate it. Attrition could be seen then as a failure to adapt to this culture without having the opportunity to resist it or change it.

Previous research has pointed out that residents who are able to craft their jobs have less intention to leave the programme, and this has been proposed as a solution for the attrition phenomenon.^45^, ^46^ Job crafting is the proactive alteration of one's work tasks, relationships and perceptions to better fit personal goals and preferences.^46^ While it may seem challenging for residents to independently redefine their roles or relationships in the hierarchical and structured environment of surgical training, job crafting literature suggests it can manifest in smaller but meaningful ways. For instance, residents may seek out specific learning opportunities, foster supportive relationships with colleagues or reframe challenging tasks to find greater personal meaning in their work. This process enables residents to adapt their roles to improve job satisfaction, engagement and well‐being. However, what is clear from our study is how challenging it is for residents who struggle to adapt to the residency demands to make such job‐crafting alterations. In fact, it is not enough to want to fit personal goals and preferences with that of the specific context—like wanting to be a ‘good surgeon’—if the culture has already decided that that is not the case. Alternatively, one can suggest that by granting residents autonomy and shaping more rewarding and value‐aligned work experiences, they might be internalising problematic elements of the sociocultural context, becoming promoters of attrition. This line of research has also proven that CT with transformational leadership can improve residents' job‐crafting abilities, decreasing attrition.^47^, ^48^, ^49^ This leadership could be a way to maximise the good elements of the ‘good surgeon’ narrative—such as prioritising empathy towards the patients—while minimising its adverse effects—such as creating an atmosphere of tension and mistreatment of the resident. However, for a cultural transformation to become a reality, these CTs need to hold better positionality to wield enough power to effectively change aspects of the culture that promote attrition.

This study is not without limitations. First, despite a thorough outreach effort, we were unable to engage with every resident who left the programme, resulting in a relatively small sample size of leavers. While this limited our ability to capture a broader range of attrition experiences, we achieved sufficient information power^50^ by maintaining a narrow study aim, a dense sample specificity and tailored interview guides informed by hermeneutic phenomenology tenets. Second, we did not include current residents in our sample, as our primary goal was to capture the institutional culture's influence on attrition from the perspective of those who had both experienced the programme as residents and now shape it as CTs. Current residents, still in the midst of their training, may not yet have the reflective perspective necessary to critically assess the cultural factors contributing to attrition, and their ongoing status in the programme may have affected the openness of their responses. However, we acknowledge that including current residents in future studies would add an important dimension, providing insight into how their experiences align with or differ from those of former residents and CTs. Finally, we were unable to fully investigate how individual characteristics, particularly gender, affect interactions within the surgical department and contribute to attrition. Recognising the importance of personal attributes like gender in shaping resident experiences, future studies could explore how these factors influence interactions with the institutional culture, particularly how female residents navigate and adapt to the environment and how these dynamics affect their decisions to stay or leave the programme.

## 5 CONCLUSION

General surgery CTs are key players in resident attrition, often unknowingly, which may shed light on the underlying causes of this issue. Attrition rates will persist unless the sociocultural drivers are addressed. It is crucial to enhance the constructive elements of the ‘good surgeon’ narrative, such as patient care commitment, while mitigating its detrimental impacts, such as fostering a stressful and harsh environment for residents. A critical step involves highlighting CTs' roles in sustaining this culture, thereby holding them accountable for driving the cultural shift needed for all residents, including those facing challenges, to successfully complete their training.

## AUTHOR CONTRIBUTIONS

**Arlen Astrid Rada‐Estarita:** Conceptualization; investigation; writing – original draft; methodology; writing – review and editing; formal analysis; data curation. **María Camila Rincón‐Ortiz:** Conceptualization; investigation; writing – original draft; methodology; writing – review and editing; formal analysis; data curation. **Oscar Geovanny Hernández‐Rodríguez:** Conceptualization; investigation; writing – original draft; methodology; writing – review and editing; formal analysis; data curation. **Francisco Manuel Olmos‐Vega:** Conceptualization; investigation; writing – original draft; methodology; writing – review and editing; formal analysis; supervision.

## CONFLICT OF INTEREST STATEMENT

The authors do not have any conflict of interest to declare.

## ETHICS STATEMENT

We received ethical approval from the institutional review board of the institution where the surgical department was part. Informed consent was requested by two of the researchers, who were free of any real or potential conflict of interest with participants, as they were not part of the department where the study took place. Likewise, these two researchers conducted interviews with the residents who dropped out of the programme to ensure their anonymity. Data were deidentified, and, given the topic's sensitive nature, we redact or decouple data that could link participants to their data, including personal details, time and chronology details and relationships within the events recounted during the interviews. These anonymised transcripts were stored in a OneDrive folder shared by the researchers and accessed through a password.

## Supporting information


**Appendix S1.** Clinical teachers' interview questions.
**Appendix S2.** Leavers' interview questions.

## Data Availability

The data that support the findings of this study are available on request from the corresponding author. The data are not publicly available due to privacy or ethical restrictions.
