# Supplementary material for: Sculpting the good surgeon or excising the bad one: How clinical teachers could perpetuate attrition in surgical residency programmes
Source: Med Educ. 2024 Oct 20;59(3):328–37. doi: 10.1111/medu.15557 (PMC11789842; doi:10.1111/medu.15557)
Supplement: Supplementary file 1 — Appendix S1. Clinical teachers' interview questions. Appendix S2. Leavers' interview questions. [file MEDU-59-328-s001.docx]

***Appendix 1***

***Personal teacher interview questions:***

***Initial questions:***

Good morning, thank you for participating in this interview and for giving us your time for this interview.

Please introduce yourself, tell us who you are.

What is your teaching role in the program and your track record in the program?

As a teacher of the specialization program in general surgery PUJ:

What motivated you to become a teacher?

How do you feel that as a teacher you have influenced the training, performance and motivation of your residents?

***Key questions***

Have you ever thought about leaving the program as a resident, please tell us your experience?

If yes, tell us what influenced or motivated you not to do so?

What has been your experience regarding residents who have decided to leaving the program?

Describe the events that led to the abandonment of any resident?

What do you think were the factors or reasons that led residents to leave the program?

What strategies were implemented within the program, or what strategies did you implement to avoid attrition?

What has been the impact of the decision of students who drop out on their clinical practice or on a personal level?

***Final***

Is there anything specific you would like to add? Or, what do you consider to be the most important thing to add from the discussion?

***Appendix 2***

***Desertors personal interview questions***

***Initial questions***

Good morning, thank you for participating in this interview and for giving us your time for this interview.

Tell us who are you, what are you doing at the moment?

What was your motivation for choosing the general surgery residency and especially the PUJ program?

***Key questions***

Tell us about your current thoughts or feelings about what happened at that time.

Tell us about a typical day in your residence before you left, what was your usual routine like?

Please make a timeline highlighting the 5 most important events that influenced your decision to leave the program.

What led to your decision to leave the PUJ general surgery program?

What was the support from hospital or university staff in the process?

What was the role of your clinical faculty in the process?

What happened in your life after the retirement of the program?

***Final questions***

What advice would you give to a person in the same situation? Or, of all the things you have discussed, what do you consider to be the most important?
